# β-catenin activity in late hypertrophic chondrocytes locally orchestrates osteoblastogenesis and osteoclastogenesis

**DOI:** 10.1242/dev.137489

**Published:** 2016-10-15

**Authors:** Astrid Houben, Daniela Kostanova-Poliakova, Martina Weissenböck, Julian Graf, Stefan Teufel, Klaus von der Mark, Christine Hartmann

**Affiliations:** 1 Institute of Experimental Musculoskeletal Medicine, Medical Faculty of the University of Münster, Domagkstrasse 3, 48149 Münster, Germany; 2 Research Institute of Molecular Pathology, Dr. Bohr-Gasse 7, A-1030 Vienna, Austria; 3 Dept. of Experimental Medicine I, University of Erlangen-Nürnberg, Glückstrasse 6, 91054 Erlangen, Germany

**Keywords:** Rankl, Tnfsf11, Tnfrsf11b, Beta-catenin, Chondrocyte-derived osteoblasts, Trabecular bone, Transdifferentiation, Mouse

## Abstract

Trabecular bone formation is the last step in endochondral ossification. This remodeling process of cartilage into bone involves blood vessel invasion and removal of hypertrophic chondrocytes (HTCs) by chondroclasts and osteoclasts. Periosteal- and chondrocyte-derived osteoprogenitors utilize the leftover mineralized HTC matrix as a scaffold for primary spongiosa formation. Here, we show genetically that β-catenin (encoded by *Ctnnb1*), a key component of the canonical Wnt pathway, orchestrates this remodeling process at multiple levels. Conditional inactivation or stabilization of β-catenin in HTCs by a *Col10a1*-Cre line locally modulated osteoclastogenesis by altering the Rankl:Opg ratio in HTCs. Lack of β-catenin resulted in a severe decrease of trabecular bone in the embryonic long bones. Gain of β-catenin activity interfered with removal of late HTCs and bone marrow formation, leading to a continuous mineralized hypertrophic core in the embryo and resulting in an osteopetrotic-like phenotype in adult mice. Furthermore, β-catenin activity in late HTCs is required for chondrocyte-derived osteoblastogenesis at the chondro-osseous junction. The latter contributes to the severe trabecular bone phenotype in mutants lacking β-catenin activity in HTCs.

## Abstract (precis)

**Summary:** The conditional modulation of β-catenin activity in late hypertrophic chondrocytes locally regulates osteoclast differentiation and the transdifferentiation of chondrocytes into osteoblasts.

## INTRODUCTION

Severe loss of trabecular bone leads to osteopenia/osteoporosis, while a gain in trabecular bone mass is termed osteopetrosis. Trabecular bone formation is the final step in endochondral bone formation during fetal development, occurring at the chondro-osseous front in conjunction with the formation of a bone marrow cavity. For this, terminally differentiated hypertrophic chondrocytes (HTCs) need to be turned over. They undergo apoptosis and are actively removed by osteoclasts and chondroclasts (Bianco et al., 1998; Shapiro et al., 2005) or, alternatively, transdifferentiate into osteoblasts (Park et al., 2015; Yang et al., 2014a,b; Zhou et al., 2014).

HTC differentiation is accompanied by extracellular matrix changes, such as type X collagen (Col10a1) production, matrix mineralization, as well as matrix remodeling due to the upregulation of matrix metalloproteinase 13 (MMP13) (Gack et al., 1995; Mattot et al., 1995). Mature HTCs also produce vascular endothelial growth factor (VEGF). VEGF induces blood vessel invasion into the transition zone between the two hypertrophic domains, facilitating bone marrow cavity formation and attracting blood vessels to the chondro-osseous front (Gerber and Ferrara, 2000; Gerber et al., 1999; Maes et al., 2002; Zelzer et al., 2004, 2002). Along with the blood vessels, precursors of osteoclasts and perichondrial-derived osteoblasts invade. The latter utilize, together with the chondrocyte-derived osteoblast precursors, the mineralized matrix remnants of late HTCs as a template for trabecular bone formation. The HTC matrix is degraded by matrix metalloproteases such as MMP13 produced by late HTCs and MT1-MMP (also known as MMP14) and MMP9 produced by osteoclasts and chondroclasts (Zhou et al., 2000; Vu et al., 1998; Inada et al., 2004; Stickens et al., 2004; Holmbeck et al., 2003). Besides VEGF, which stimulates osteoclast recruitment, HTCs produce factors such as osteopontin (Opn; also known as Spp1), receptor activator of nuclear factor kappa-B ligand (Rankl; also known as Tnfsf11) and its antagonist osteoprotegerin (Opg; also known as Tnfrsf11b) that regulate osteoclastogenesis at the chondro-osseous front (Kishimoto et al., 2006; Nakashima et al., 2011; Silvestrini et al., 2005; Usui et al., 2008; Xiong et al., 2011). Osteoclast activity, and in particular MMP9, has been proposed to facilitate the release of matrix-bound bioactive VEGF (Bergers et al., 2000; Colnot et al., 2003; Engsig et al., 2000; Ortega et al., 2010).

The Wnt/β-catenin pathway regulates skeletogenesis at many levels, including by influencing cell lineage decisions (Day et al., 2005; Hill et al., 2005; Spater et al., 2006) and cell differentiation within the perichondrium-derived osteoblast lineage (Rodda and McMahon, 2006) or the chondrocyte lineage (Tamamura et al., 2005). The cytosolic levels of β-catenin determine pathway activity. Activation by a Wnt ligand results in increased cytosolic β-catenin levels and its subsequent translocation into the nucleus, where it acts as a transcriptional coactivator (MacDonald et al., 2009). Continuous Wnt/β-catenin signaling in immature chondrocytes leads to their dedifferentiation and blocks hypertrophy (Guo et al., 2004; Miclea et al., 2009; Tamamura et al., 2005). Inducible β-catenin activation in chondrocytes *in vivo* and β-catenin activation or *Wnt9a* overexpression in more mature chondrocytes *in vitro* stimulates hypertrophy and expression of the late hypertrophic markers *Mmp13* and *Vegfa*, while slightly decreasing *Col10a1* levels (Dao et al., 2012; Tamamura et al., 2005; Chen et al., 2008). Moreover, in perichondrium-derived osteoblasts the pathway postnatally influences bone homeostasis (Glass et al., 2005; Holmen et al., 2005; Kramer et al., 2010; Sato et al., 2009; Spencer et al., 2006). A recent study associated β-catenin signaling in *Col2a1*-expressing chondrocytes with the regulation of postnatal bone development (Wang et al., 2014).

Previous experimental settings might have obscured the requirement for β-catenin in differentiated HTCs. Hence, we specifically modulated β-catenin function in HTCs using a *Col10a1*-Cre line (Gebhard et al., 2008) and either conditionally deleted the *Ctnnb1* gene or stabilized β-catenin at the protein level. Recently, *Ctnnb1* deletion from HTCs has been shown to result postnatally in decreased trabecular bone density due to a local increase in osteoclast number through upregulation of *Rankl* (Golovchenko et al., 2013). Here, we show that this phenotype is already present in the embryo and that the augmented osteoclastogenesis as a result of increased Rankl does not fully account for the phenotype, as decreasing *Rankl* expression specifically in HTCs only partially reverts the phenotype. Our analysis uncovered an additional need for β-catenin in the differentiation of HTC-derived osteoblasts. Stabilization of β-catenin in HTCs, by contrast, interfered with late HTC turnover, affecting embryonic and postnatal bone marrow formation. The embryonic phenotype was associated with a reduction in osteoclast number due to reduced *Rankl* expression and could be reverted in part by additional removal of *Opg.* In the long bones of adult mice, HTC-derived osteoblast differentiation was promoted at the more active growth plates.

## RESULTS

### Loss of β-catenin activity from HTCs results in reduced trabecular bone formation

**Fig. 1.**
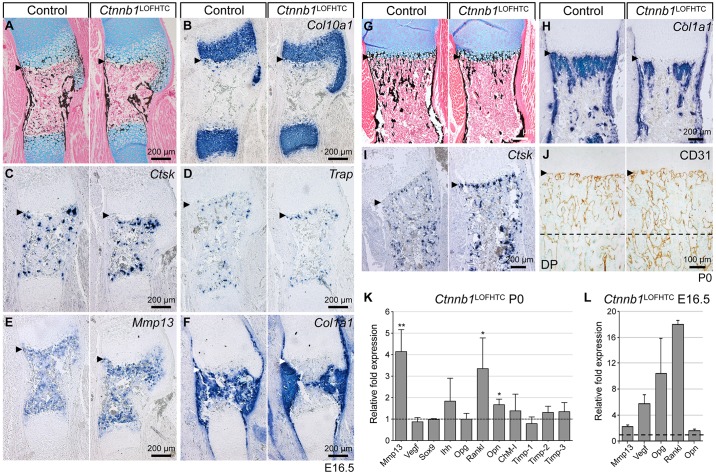
**Phenotypic analysis of *Ctnnb1*^LOFHTC^ mouse mutants.** (A-F) Representative images of alternating sections through humeri of E16.5 control and *Ctnnb1*^LOFHTC^ mutant littermates. (A) Alcian Blue/von Kossa staining. (B) Hypertrophic zones visualized by *Col10a1 in situ* hybridization (ISH). (C) *Ctsk* ISH. (D) *Trap*-positive osteoclasts visualized by ISH. (E) *Mmp13* ISH. (F) *Col1a1* ISH. (G-J) Representative alternating sections through humeri of P0 control and *Ctnnb1*^LOFHTC^ mutant littermates. (G) Alcian Blue/von Kossa. (H) *Col1a1* ISH. (I) *Ctsk* ISH. (J) Visualization of the vascular network by CD31 (Pecam1) immunostaining. Arrowheads point to the chondro-osseous border. The dashed line indicates the beginning of the diaphysis (DP). (K) qPCR analysis of P0 *Ctnnb1*^LOFHTC^ relative to control long bone material (*n*=3). *P<0.05, **P<0.01. Genotype of control in A-K is *Ctnnb1*^fl/+^;*Col10a1*-Cre^−^. (L) qPCR analysis using E16.5 YFP sorted material from *Ctnnb1*^LOFHTC^;*Rosa*^YFP/+^ long bones relative to material isolated from E16.5 *Col10a1*-Cre^+^;*Rosa*^YFP/+^ limbs (*n*=2). Error bars indicate s.d.

Consistent with a previous report, conditional *Ctnnb1* inactivation from HTCs using *Col10a1*-Cre resulted in viable mice (*Ctnnb1*^l^^acZ/fl^;*Col10a1*-Cre^+^, hereafter *Ctnnb1*^LOFHTC^) with an osteopenic phenotype (Golovchenko et al., 2013). The phenotype was analyzed from embryonic day (E) 16.5 onwards, focusing primarily on the humerus. At this stage, the primary spongiosa begins to form in the wild type. Alcian Blue and von Kossa staining revealed a severe reduction of calcified structures in the bone marrow cavity of *Ctnnb1*^LOFHTC^ mutants (Fig. 1A). Based on histology and *Col10a1 in situ* staining, the hypertrophic domains were not altered in size by the loss of *Ctnnb1* (Fig. 1A,B). *In situ* hybridization for the osteoclast markers cathepsin K (*Ctsk*) and tartrate-resistant acid phosphatase (*Trap*; also known as *Acp5*) revealed an accumulation of cells positive for *Ctsk* and *Trap* at the chondro-osseous front (Fig. 1C,D). Staining for *Mmp13* and *Opn*, which are both expressed in terminal mature HTCs and osteoblasts, appeared slightly increased at the chondro-osseous front in *Ctnnb1*^LOFHTC^ embryos (Fig. 1E, Fig. S1H). By contrast, staining for the osteoblastic marker *Col1a1* was restricted to the central region of the maturation zone, aside from its strong periosteal expression (Fig. 1F).

Histology and marker expression in E18.5 and postnatal day (P) 0 humeri was similar to that at E16.5, showing reduced trabeculation, reduction in *Col1a1*-positive structures and an increase in cells positive for osteoclast markers at the chondro-osseous front (Fig. 1G-I, Fig. S1A-F). *Mmp9*-positive osteoclasts/chondroclasts were also increased in number at the chondro-osseous front (Fig. S1G). Staining for the prehypertrophic marker Indian hedgehog (*Ihh*) appeared to be slightly more intense (Fig. S1D). CD31 (also known as Pecam1) staining for endothelial cells revealed no apparent differences in vascularization close to the chondro-osseous front, but hypervascularity was apparent in the diaphysis (Fig. 1J). *Vegf* (*Vegfa*) expression in mature HTCs appeared to be similar to the control at E18.5 (Fig. S1C). Quantitative PCR (qPCR) analysis at P0 (Fig. 1K) and E16.5 (Fig. 1L) revealed that *Rankl* transcript levels were increased in *Ctnnb1*^LOFHTC^ mutants compared with controls at both time points, as were *Mmp13* and *Opn* transcript levels (Fig. 1K,L). Surprisingly, *Vegf* and *Opg* levels were both increased at E16.5, but not significantly altered at P0 (Fig. 1K,L). *Ihh* expression showed a slight, but not statistically significant, increase at P0 (Fig. 1K).

In summary, inactivation of β-catenin in HTCs results in an increase in osteoclast number, loss of mineralized structures and increased expression of *Mmp13*, *Opn* and *Rankl* – factors that promote osteoclastogenesis.

### Stabilization of β-catenin in HTCs results in an expansion of the mineralized hypertrophic zone

**Fig. 2.**
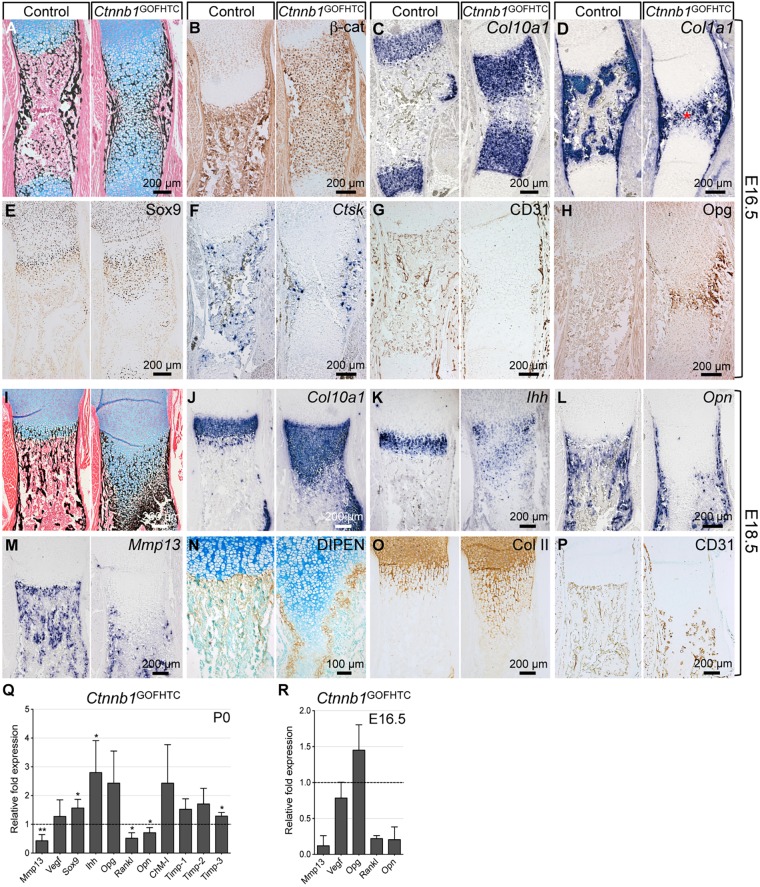
**Phenotypic analysis of E16.5 and E18.5 *Ctnnb1*^GOFHTC^ mutants.** (A-H) Representative images of alternating sections through humeri from E16.5 *Ctnnb1*^GOFHTC^ and control (*Ctnnb1*^ex3fl/+^;*Col10a1-*Cre^−^) littermates. (A) Alcian Blue/von Kossa staining. (B) β-catenin immunohistochemical staining. (C) *Col10a1* ISH. (D) *Col1a1* ISH showing the presence of chondrocyte-like, *Col1a1*-positive cells (red asterisk). (E) Sox9 immunohistochemical staining. (F) *Ctsk* ISH for osteoclasts. (G) CD31 immunohistochemical staining for blood vessels. (H) Opg immunohistochemical staining. (I-P) Representative images of E18.5 humerus sections of *Ctnnb1*^GOFHTC^ and control (*Ctnnb1*^ex3fl/+^;*Col10a1-*Cre^−^) littermates. (I) Histological changes visualized by Alcian Blue/von Kossa staining. (J-M) ISH for *Col10a1* (J), *Ihh* (K), *Opn* (L) and *Mmp13* (M). (N) Immunohistochemical staining for the aggrecan neoepitope DIPEN, counterstained with Methyl Green. (O) Immunohistochemical staining for type II collagen (Col II). (P) Immunohistochemical staining for CD31. (Q) qPCR analysis of P0 *Ctnnb1*^GOFHTC^ relative to control long bone material (*n*=3) ±s.d. *P<0.05, **P<0.01. (R) qPCR analysis using E16.5 YFP sorted material from *Ctnnb1*^GOFHTC^;*Rosa*^YFP/+^ long bones relative to material isolated from E16.5 *Col10a1*-Cre^+^;*Rosa*^YFP/+^ limbs (*n*=2).

Conditional stabilization of β-catenin in HTCs using *Col10a1*-Cre (*Ctnnb1*^ex3fl/+^;*Col10a1*-Cre^+^, hereafter *Ctnnb1*^GOFHTC^) revealed a massive expansion of the two mineralized HTC zones in E16.5 humeri, which remained connected (Fig. 2A). In the mutant, high levels of β-catenin were detected in all HTCs throughout the expanded zone (Fig. 2B). At the molecular level, the two *Col10a1* expression domains were expanded in the mutant, but did not connect (Fig. 2C). In the centralmost region, where the cells still appeared to be chondrogenic in nature based on their rounded morphology and Alcian Blue-positive matrix, only a few cells expressed *Col10a1* (Fig. 2C). These rounded, chondrocyte-like cells expressed *Col1a1*, which is not normally expressed by HTCs but rather by osteoblast precursors and mature osteoblasts, as seen in the control (Fig. 2D). The expression of *Col10a1* and *Col1a1* in the expanded HTC zone in the mutants was nearly mutually exclusive (Fig. S2A,C). These *Col1a1*-expressing chondrocyte-like cells also expressed the osteoblast marker parathyroid hormone 1 receptor (*Ppr*; also known as *Pth1r*) (Fig. S2B), but not the more mature osteoblast marker osteocalcin (also known as *Bglap*) (data not shown). Sox9 protein persisted in the expanded hypertrophic zone of the mutants (Fig. 2E). In the *Ctnnb1*^GOFHTC^ mutants, osteoclasts positive for *Ctsk* and *Mmp9* were restricted to the bone collar region and reduced in number (Fig. 2F; data not shown). Accordingly, blood vessel invasion visualized by CD31 staining was compromised in *Ctnnb1*^GOFHTC^ mutants (Fig. 2G). Cells in the central region stained intensely for Opg (Fig. 2H). Analysis of histology and molecular markers in E18.5 mutant humeri revealed very similar results to E16.5 (Fig. 2I-P; data not shown). The *Col10a1* and *Ihh* domains were expanded (Fig. 2J,K). Interestingly, late HTC markers such as *Opn* and *Mmp13* were not expressed in the expanded hypertrophic zone (Fig. 2L,M). Staining for the aggrecan neoepitope DIPEN (Fosang et al., 1996), which was absent in the expanded region, together with a concurrent expansion of the collagen type II-positive region (Fig. 2N,O), confirmed the lack of Mmp13 in the central core. DIPEN-positive cells were only present at the edges of the expanded HTC zone and the two surrounding rows of cells, which by morphology appeared chondrocyte-like (Fig. 2N). Blood vessel invasion had proceeded but still appeared different to the control (Fig. 2P).

qPCR using mRNA from P0 skeletal elements (Fig. 2Q) and E16.5 sorted chondrocytes (Fig. 2R) revealed that, upon stabilization of β-catenin, *Mmp13*, *Opn* and *Rankl* levels were decreased, whereas *Ihh* and *Sox9* levels were slightly increased, as were *Opg* levels but, to our surprise, not all cells that produced stabilized β-catenin produced Opg (Fig. 2H). Although the overall phenotype of *Ctnnb1*^GOFHTC^ mutants resembled that of transgenic animals expressing *Sox9* under the control of the *Col10a1* promoter (Hattori et al., 2010), we did not observe downregulation of *Vegf*; instead, the levels of anti-angiogenic factors such as chondromodulin 1 (*ChM-I*; also known as *Lect1*) and tissue inhibitor of metalloproteinase 1-3 (*Timp1-3*), were increased (Fig. 2Q). In addition to their function as metalloproteinase inhibitors, Timp2 and Timp3 can directly inhibit VEGF signaling (Qi et al., 2003; Seo et al., 2003). The upregulation of these anti-angiogenic factors might contribute to the failure of blood vessels to invade the hypertrophic region.

**Fig. 3.**
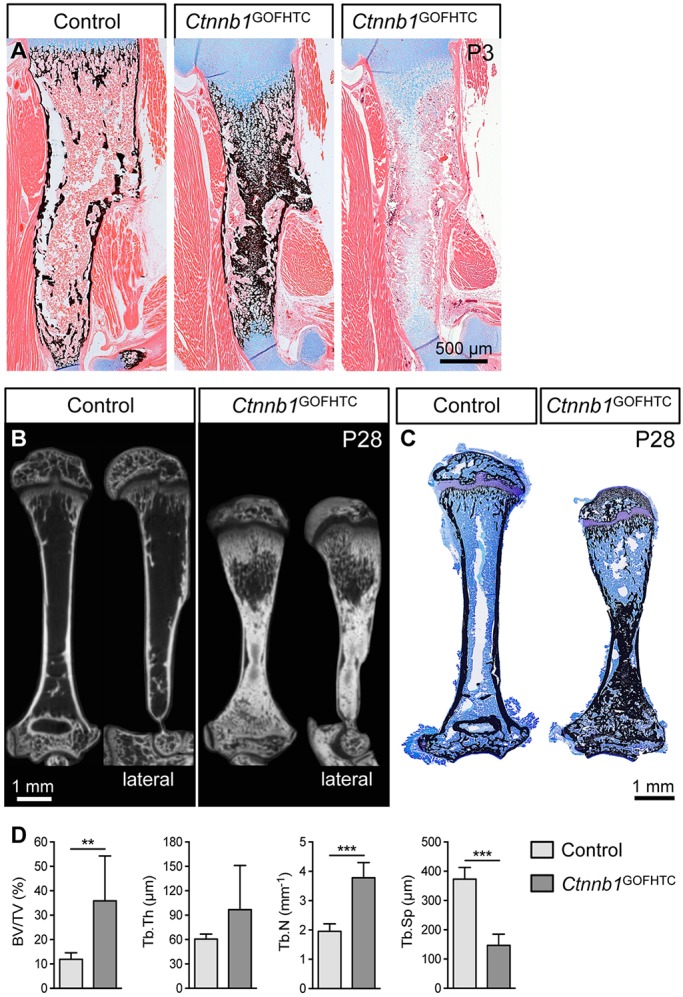
**Postnatal phenotypic analysis of *Ctnnb1*^GOFHTC^ mutants.** (A) Representative images of humeri from P3 control and *Ctnnb1*^GOFHTC^ littermates stained with Alcian Blue/von Kossa or Alcian Blue. (B) MicroCT images of control and *Ctnnb1*^GOFHTC^ humeri (P28, frontal and lateral view). The lateral view reveals anterior-posterior differences in the trabecular density underneath the proximal growth plate in the mutant humerus. (C) Toluidine Blue/von Kossa-stained sections of control and *Ctnnb1*^GOFHTC^ P28 humeri. Orientation is proximal up and distal down. (D) Histomorphometric quantification of the bone volume (BV) to total volume (TV) ratio, trabecular thickness (Tb.Th), trabecular number (Tb.N) and trabecular spacing (Tb.Sp) of P28 proximal humeri comparing *Ctnnb1*^GOFHTC^ (*n*=8) and littermate controls (*n*=9). **P<0.01, ***P<0.001. Error bars indicate s.d. Genotype of control is *Ctnnb1*^ex3fl/+^;*Col10a1-*Cre^−^.

We next asked whether the mineralized HTCs would be removed after birth. In the long bones of P3 mutant pups the mineralized hypertrophic zones of the proximal and distal growth plates were still expanded and connected (Fig. 3A). The mutant long bones were slightly shorter. This shortening was more pronounced in P28 mice, which overall were smaller than their littermate controls (*Ctnnb1*^ex3fl/+^; *Col10a1-*Cre^−^) (Fig. S3A,B). MicroCT-analysis and histological examination of 4-week-old humeri and femora revealed a locally restricted osteopetrotic phenotype in *Ctnnb1*^GOFHTC^ compared with littermate controls (Fig. 3B,C, Fig. S3C). Closer examination uncovered differences between distal and proximal regions of the skeletal elements: in the humerus, bone marrow space was present proximally but almost completely replaced by abnormally mineralized tissue distally (Fig. 3B,C). In the femur this pattern was reversed (Fig. S3C). Interestingly, bone remodeling and the formation of bone marrow space was always associated with the side of the growth plate that has been described in humans to be the more active, e.g. the proximal humeral and the distal femoral growth plate (Fig. 3B,C, Fig. S3C; data not shown) (Pritchett, 1991, 1992). The mineralized HTC zone was increased in the proximal growth plates of humeri and femora and the distal femoral growth plate (Fig. 3C, Fig. S3C). Furthermore, in the proximal humeri and distal femora, trabecular bone volume with respect to total volume (BV/TV) and trabecular number were increased and, accordingly, trabecular spacing was decreased, while trabecular thickness was unaltered (Fig. 3D, Fig. S3D).

In summary, with the exception of the active growth plates in juveniles, stabilization of β-catenin in HTCs interferes with the removal of mineralized HTCs and blood vessel invasion. This is associated with the downregulation of pro-osteoclastic factors, a reduced number of osteoclasts and the upregulation of anti-angiogenic factors.

### Genetic alteration of osteoclastogenesis partially rescues phenotypic changes caused by loss or stabilization of β-catenin in late HTCs

**Fig. 4.**
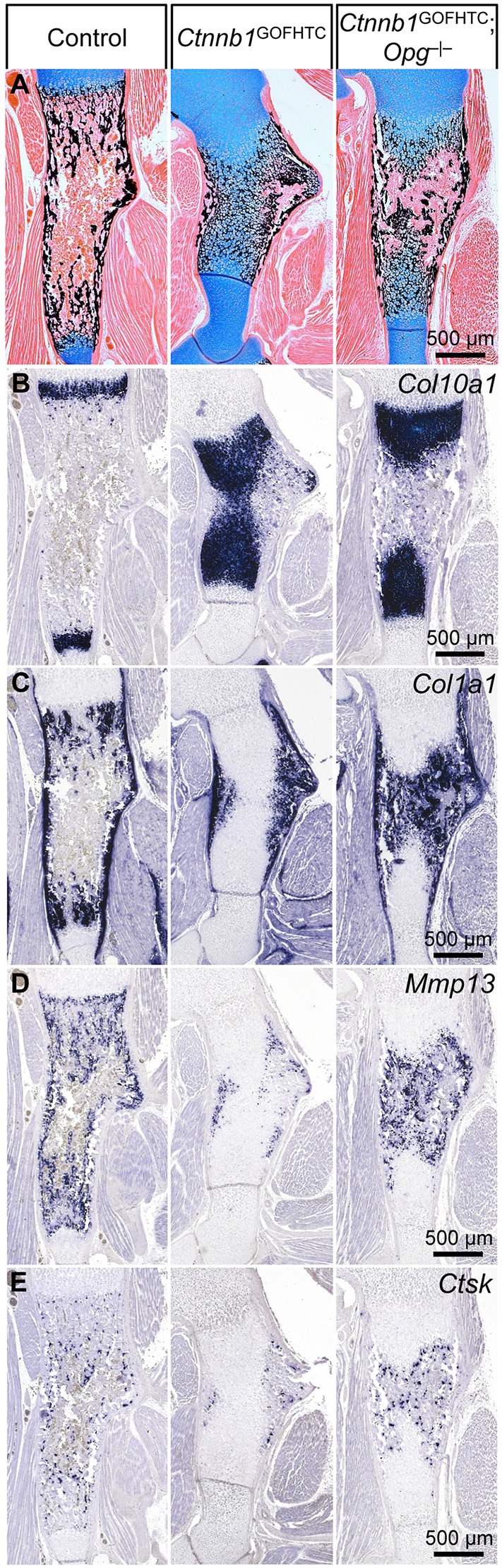
**Partial rescue** **of the *Ctnnb1*^GOFHTC^ phenotype by the loss of Opg.** (A-E) Representative images of sections through E19.5 humeri of control (*Ctnnb1*^ex3fl/+^;*Col10a1-*Cre^−^), *Ctnnb1*^GOFHTC^ and *Ctnnb1*^GOFHTC^;*Opg*^−/−^ specimens. (A) Histological changes visualized by Alcian Blue/von Kossa staining showing remodeling of the extended zone of mineralized HTCs in the *Ctnnb1*^GOFHTC^ upon loss of *Opg*. (B-E) ISH for *Col10a1* (B), *Col1a1* (C), *Mmp13* (D) and *Ctsk* (E).

Conditional loss or stabilization of β-catenin in late HTCs led to an alteration in expression of osteoclastogenesis modulating factors, such as *Opg* and *Rankl*, and as a result altered the location and/or number of osteoclasts. Using a genetic approach we examined whether local downregulation or increased activation of osteoclastogenesis can rescue the phenotypic changes that occur upon conditional loss or stabilization of β-catenin. Examination of E19.5 *Ctnnb1*^GOFHTC^;*Opg*^−/−^ double-mutant humeri (*Ctnnb1*^ex3fl/+^;*Col10a1*-Cre^+^;*Opg*^−/−^) revealed a partially rescued phenotype in all specimens examined (5/5) (Fig. 4). Bone marrow space was restored but trabecular bone formation was still abnormal and the mineralized HTC zones were still enlarged (Fig. 4A,B). *Col1a1* and *Mmp13* staining revealed the presence of osteoblasts and *Ctsk* staining the presence of osteoclasts in the bone marrow of *Ctnnb1*^GOFHTC^;*Opg*^−/−^ humeri (Fig. 4C-E). Unlike in the *Ctnnb1*^LOFHTC^ mutants, no pronounced reduction of trabeculae was observed in *Opg* mutants at P0 (Fig. S4A). According to the *Col10a1* and *Ctsk* staining, the hypertrophic zone appeared normal and osteoclast numbers appeared not to be substantially increased (Fig. S4A).

**Fig. 5.**
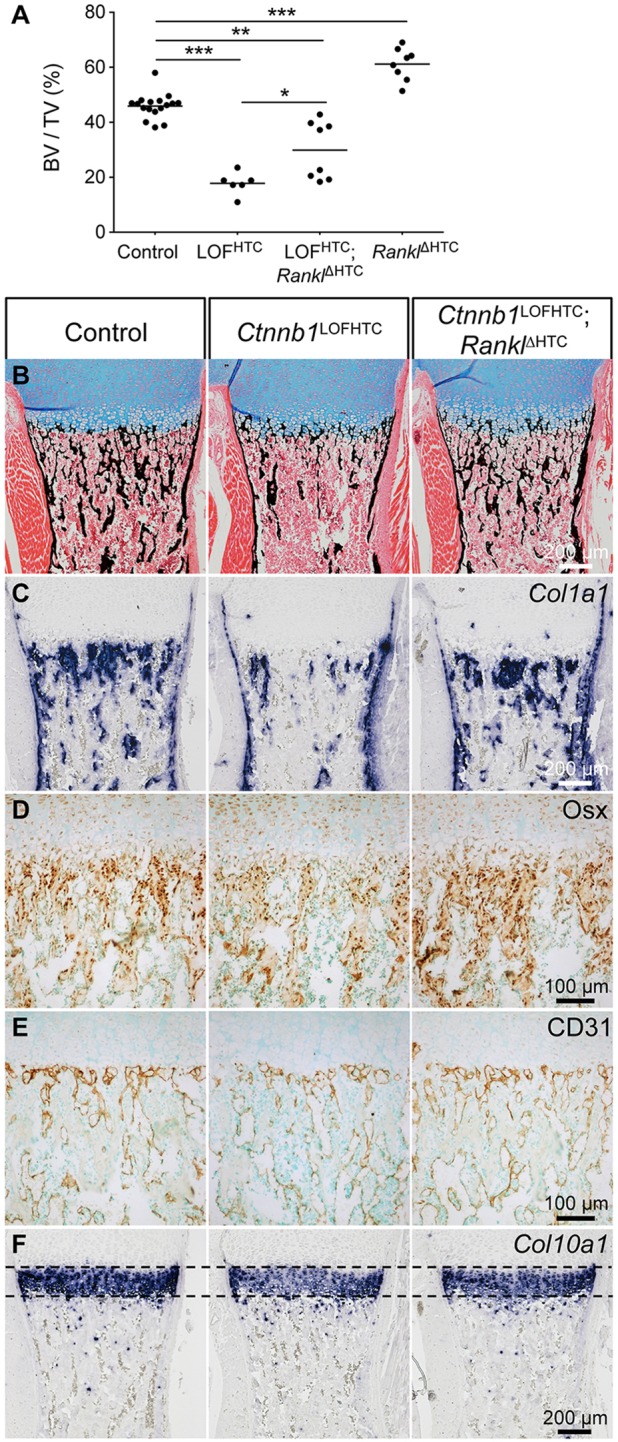
**Partial reversal of the *Ctnnb1*^LOFHTC^ phenotype by conditional deletion of *Rankl* in HTCs.** (A) Scatter plot showing the distribution (and mean) of the bone volume (BV) to total volume (TV) (%) of the different controls (*n*=17), *Ctnnb1*^LOFHTC^ (*n*=6), *Ctnnb1*^LOFHTC^;*Rankl*^ΔHTC^ (*Ctnnb1*^l^^acZ/fl^;*Rankl*^fl/fl^;*Col10a1-*Cre^+^) (*n*=8) and *Rankl*^ΔHTC^ (*Rankl*^fl/fl^;*Col10a1*-Cre^+^) (*n*=8) specimens. *P<0.05, **P<0.01, ***P<0.001. (B-F) Representative images of sections through control, *Ctnnb1*^LOFHTC^ and ‘complete’ rescue *Ctnnb1*^LOFHTC^;*Rankl*^ΔHTC^ humeri. (B) Alcian Blue/von Kossa staining. (C) *Col1a1* ISH. (D) Osx immunohistochemical staining. (E) CD31 immunohistochemical staining. (F) *Col10a1* ISH.

Given our hypothesis that β-catenin activity represses *Rankl* expression in HTCs, we decided to downregulate *Rankl* specifically in HTCs in the *Ctnnb1* mutant background. Control, *Ctnnb1*^LOFHTC^ and conditional *Ctnnb1* and *Rankl* double mutants (*Ctnnb1*^l^^acZ/fl^;*Rankl*^fl/fl^;*Col10a1*-Cre^+^, hereafter *Ctnnb1*^LOFHTC^;*Rankl*^ΔHTC^) were examined by microCT (see Fig. S5A). BV/TV measurements revealed that trabecular bone formation was restored to a variable degree in the *Ctnnb1*^LOFHTC^;*Rankl*^ΔHTC^ specimens and that this restoration was statistically significant (Fig. 5A). Based on the microCT data, the eight *Ctnnb1*^LOFHTC^;*Rankl*^ΔHTC^ specimens fell into two groups: four with an almost ‘complete’ phenotypic rescue and four with a very ‘local rescue effect’ restricted to the chondro-osseous front (Fig. 5A, Fig. S5A). The average BV/TV ratio for the four ‘complete’ rescued specimens increased to 39.56±2.40% as compared with ∼18% in the *Ctnnb1*^LOFHTC^ mutants, but was still at the low end of the range of controls with a BV/TV ratio of 45.90±4.51% (Fig. S5B). Histological and osteoblast marker analysis confirmed the variability of the rescue effect (Fig. 5B-F; data not shown). In ‘complete’ *Ctnnb1*^LOFHTC^;*Rankl*^ΔHTC^ mutants increased trabeculae numbers and an increase in *Col1a1*-positive cells and trabeculae-associated cells positive for osterix (Osx; also known as Sp7) were observed (Fig. 5B-D). Local modulation of Rankl levels did not alter the vascular pattern (Fig. 5E). However, local loss of Rankl in the *Ctnnb1*^LOFHTC^;*Rankl*^ΔHTC^ specimens decreased the overall osteoclast number compared with *Ctnnb1*^LOFHTC^ littermates (Fig. S5C). The BV/TV ratio in *Rankl*^ΔHTC^ (*Rankl*^fl/fl^;*Col10a1*-Cre^+^) newborns was increased to 61.16±5.89% (Fig. 5A, Fig. S5A,B), but no obvious decrease in *Ctsk*-stained cells was observed (Fig. S5C). In contrast to previous reports in adult mice (Nakashima et al., 2011; Xiong et al., 2011), the width of the hypertrophic zone was not altered upon deletion of *Rankl* in HTCs (Fig. 5F, Fig. S5C). In mutants lacking the receptor *Rank* (also known as *Tnfrsf11a*), no osteoclasts are present (Fig. S4B; data not shown), while some *Mmp9*-positive cells can still be detected primarily at the chondro-osseous border (Fig. S4B). Here, the *Col10a1* staining revealed a noticeable enlargement of the HTC zone (Fig. S4B). Nevertheless, in contrast to the *Ctnnb1*^GOFHTC^ mutants, remodeling of the hypertrophic zone was only slightly affected and bone marrow cavity formation was normal (compare Fig. S4B with Fig. 2I,J).

Taken together, genetic modulation of osteoclastogenesis is able to partially revert the β-catenin loss- and gain-of-function phenotypes, demonstrating that the alterations in osteoclast numbers are in part responsible for the phenotypic changes.

### β-catenin activity in late HTCs is required for the differentiation of chondrocyte-derived osteoblasts

**Fig. 6.**
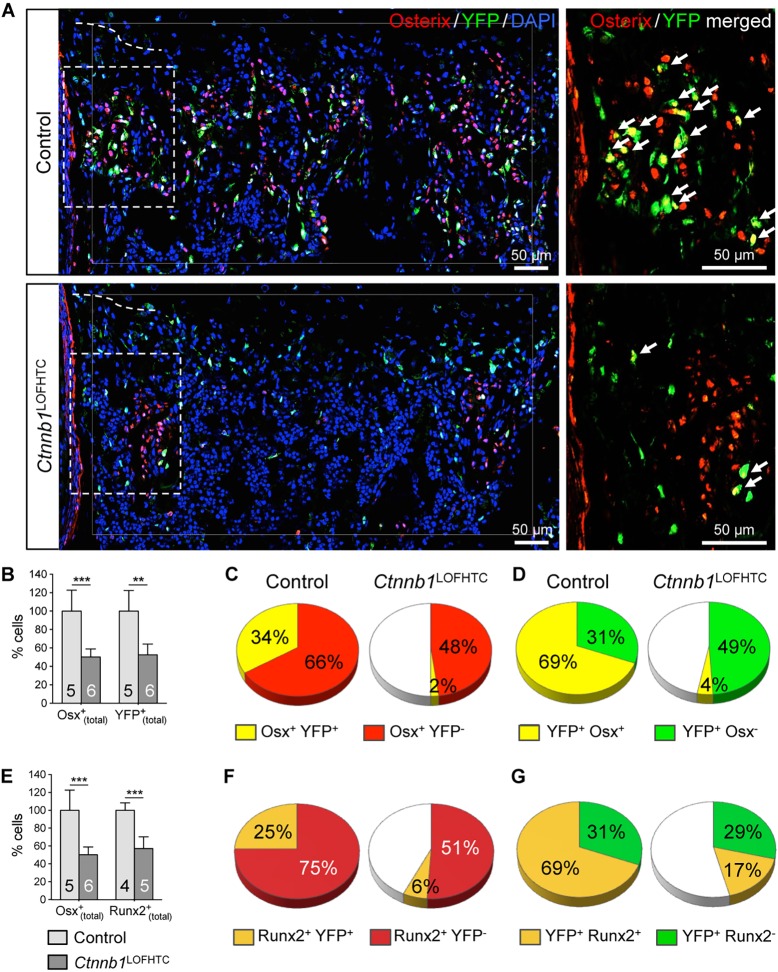
**Chondrocyte-derived osteoblastogenesis is affected by the loss of *Ctnnb1* from HTCs.** (A) Representative images of control and *Ctnnb1*^LOFHTC^ P0 specimens taken from the region below the chondro-osseous front. Immunofluorescent staining for Osx (red) and YFP (green), and DAPI staining for nuclei (blue). The dashed line indicates the position of the chondro-osseous border. The increase in DAPI-stained cells in the *Ctnnb1*^LOFHTC^ mutant reflects an increase in cellularity. Higher magnification images of the boxed regions with only the Osx (red) and YFP (green) signals are provided on the right, with double-positive cells marked by arrows. (B) Mean percentage of Osx^+^_(total)_ and YFP^+^_(total)_ cells relative to control. (C) The distribution of Osx^+^ YFP^−^ (perichondrial-derived) and Osx^+^ YFP^+^ (chondrocyte-derived) cells normalized with respect to the Osx^+^_(total)_ population (100%) of the control. (D) The distribution of YFP^+^ Osx^+^ (osteoblastic) and YFP^+^ Osx^−^ (non-osteoblastic) cells normalized with respect to the YFP^+^_(total)_ population (100%) of the control. (E) The mean percentage of Osx^+^_(total)_ and Runx2^+^_(total)_ cells relative to control. (F) The distribution of Runx2^+^ YFP^−^ (perichondrial-derived) and Runx2^+^ YFP^+^ (chondrocyte-derived) cells normalized with respect to the Runx2^+^_(total)_ population (100%) of the control. (G) The distribution of YFP^+^ Runx2^+^ (osteoblastic) and YFP^+^ Runx2^−^ (non-osteoblastic) cells normalized with respect to the YFP^+^_(total)_ population (100%) of the control. Cells for B-D were counted within the region indicated by the thin white line in A, and for E-G in a square of the same size on three or four sections per specimen and genotype. Control is *Ctnnb1*^fl/+^;*Col10a1*-Cre^+^;*Rosa*^YFP/+^; *Ctnnb1*^LOFHTC^ is *Ctnnb1*^l^^acZ/fl^;*Col10a1*-Cre ^+^;*Rosa*^YFP/+^. (B,E) **P<0.01, ***P<0.001. The number of specimens analyzed (*n*) is indicated within the bars. Error bars indicate s.d.

Given that the phenotypic rescue achieved by deleting *Rankl* in HTCs in the *Ctnnb1*^LOFHTC^ mutant background was not 100%, and that β-catenin plays a role in periosteal osteoblast differentiation (Day et al., 2005; Hill et al., 2005), as well as in the light of recent publications demonstrating that HTC descendants can differentiate into osteoblasts (Yang et al., 2014a,b; Zhou et al., 2014), we addressed the question of whether chondrocyte-derived osteoblastogenesis is altered in *Ctnnb1*^LOFHTC^ mutants. We examined *Ctnnb1*^LOFHTC^;*Rosa*^YFP/+^ humeri and determined the percentage of all osteoblasts [Osx^+^_(total)_], all chondrocyte-derived cells [YFP^+^_(total)_], of chondrocyte-derived (Osx^+^ YFP^+^) and perichondrial-derived (Osx^+^ YFP^−^) osteoblasts, as well as the osteogenic (YFP^+^ Osx^+^) and non-osteogenic (YFP^+^ Osx^−^) subpopulations of YFP^+^ cells in the subchondral growth plate region compared with littermate controls (Fig. 6).

The overall cellularity (DAPI^+^ cells) was increased in the *Ctnnb1*^LOFHTC^ mutant bone marrow (Fig. 6A). The Osx^+^_(total)_ and YFP^+^_(total)_ populations were both reduced by ∼50% compared with controls (Fig. 6B). The perichondrial-derived Osx^+^ YFP^−^ population decreased by 18%, and the chondrocyte-derived Osx^+^ YFP^+^ population dropped from 34% in the controls to 2% in *Ctnnb1*^LOFHTC^ mutants (Fig. 6C). By contrast, the non-osteoblastic YFP^+^ Osx^−^ population increased by 18%, whereas only 4% of all YFP^+^ cells differentiated towards the osteoblastic lineage (YFP^+^ Osx^+^) in the mutant (Fig. 6D).

These findings were corroborated by the analysis of Runx2 (also known as Cbfa1), a second osteoblastic marker, which acts upstream of Osx in osteoblastogenesis (Nakashima et al., 2002). Compared with the control, the Runx2^+^_(total)_ population dropped by 44% (Fig. 6E), the chondrocyte-derived Runx2^+^ YFP^+^ population from 25% to 6%, and the perichondrial-derived Runx2^+^ YFP^−^ population by 24% in the *Ctnnb1*^LOFHTC^ mutants (Fig. 6F). Interestingly, unlike the non-osteogenic YFP^+^ Osx^−^ population, the YFP^+^ Runx2^−^ population did not increase and the osteogenic YFP^+^ Runx2^+^ subpopulation did not decrease to the same extent as the YFP^+^ Osx^+^ population (Fig. 6D,G). The fact that osteoblastic chondrocyte-derived bone marrow cell numbers decreased dramatically in the long bones lacking β-catenin activity in late HTCs suggests that β-catenin is required for the differentiation of chondrocyte-derived osteoblasts and that the remaining osteoblasts are essentially all periosteal derived.

Next, we asked whether this decrease in chondrocyte-derived osteoblast number is due to the fact that the precursor cells upregulated Sox9, similar to what is observed in perichondrial-derived osteoblasts that lack β-catenin (Hill et al., 2005). Sox9 protein expression and levels were similar to the control (Fig. S6). Furthermore, no increase in TUNEL-positive or cleaved caspase 3-positive cell numbers was observed in the growth plate or at the chondro-osseous front in *Ctnnb1*^LOFHTC^ mutants (Fig. S6; data not shown). According to Ki67 staining, proliferation in the growth plate or of YFP^+^ cells was not altered (Fig. S6).

We then investigated whether the increased number of non-osteoblastic YFP^+^ Osx^−^ cells is due to a cell lineage shift into adipocytes. Adipocytes were increased in number in 4-month-old *Ctnnb1*^LOFHTC^ mutant tibiae (Fig. S7A). Similarly, the overall number of fatty acid-binding protein 4 (FABP4)-positive cells was increased in newborn *Ctnnb1*^LOFHTC^ limbs compared with controls (Fig. S7B). However, none of the FABP4^+^ cells, located in close proximity to blood vessels, stained positively for YFP (Fig. S7B-D). Thus, we conclude that the increase in adipocyte number is not caused by a lineage shift of chondrocyte-derived osteoblast precursors.

### Increased β-catenin activity promotes transdifferentiation of chondrocyte-derived osteoblasts

**Fig. 7.**
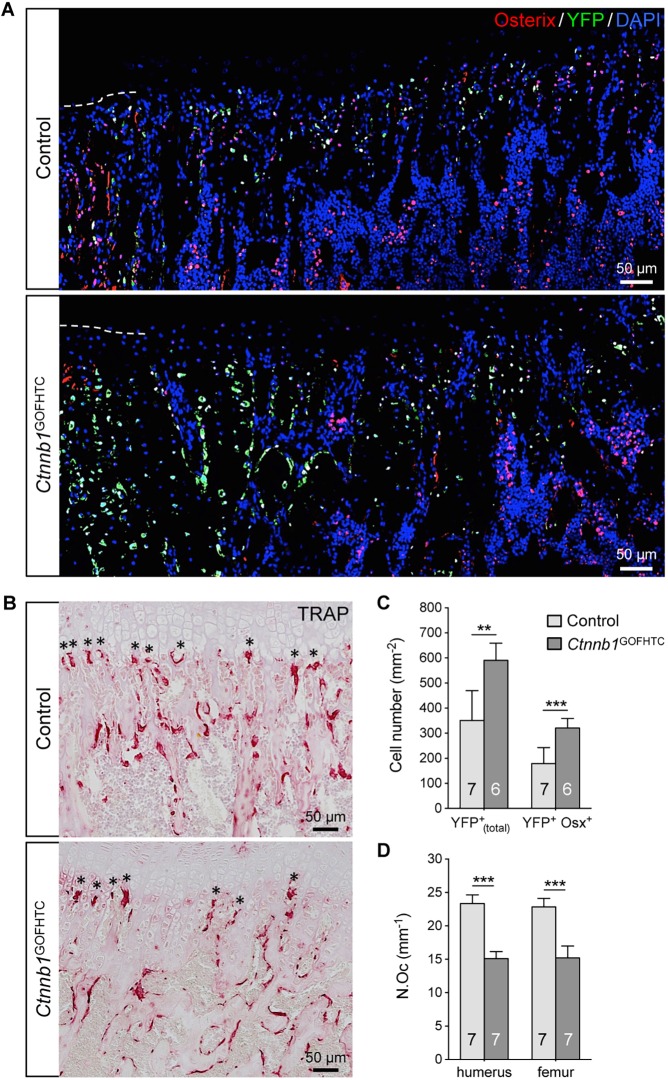
**Alteration in chondrocyte-derived osteoblast and osteoclast numbers in P28 *Ctnnb1*^GOFHTC^ specimens.** (A) Representative images of control and *Ctnnb1*^GOFHTC^ specimens below the chondro-osseous front at the proximal humeral growth plate. Immunofluorescent staining for Osx (red) and YFP (green), and DAPI staining for nuclei (blue). The dashed line indicates the position of the chondro-osseous border. (B) TRAP staining on control and *Ctnnb1*^GOFHTC^ littermates. Asterisks mark the osteoclasts at the chondro-osseous border. (C) The mean percentage of YFP^+^_(total)_ (which are progeny of *Col10a1*-Cre-expressing HTCs) and YFP^+^ Osx^+^ (the chondrocyte-derived osteogenic population) cells determined within the area 250 µm below the chondro-osseous front. (D) The mean number of osteoclasts lining the chondro-osseous front (marked by asterisks in B) in humerus and femur of control and *Ctnnb1*^GOFHTC^ specimens. Control is *Col10a1*-Cre^+^;*Rosa*^YFP/+^; *Ctnnb1*^GOFHTC^ is *Ctnnb1^ex3^*^fl/+^;*Col10a1*-Cre^+^;*Rosa*^YFP/+^. (C,D) **P<0.01, ***P<0.001. The number of samples analyzed (*n*) is indicated within the bars. Error bars indicate s.d.

Finally, we analyzed whether increased β-catenin activity in late HTCs affects the number of chondrocyte-derived osteoblasts. Counting the YFP^+^ as well as the YFP^+^ Osx^+^ cells in an area spanning 250 µm below the chondro-osseous border (Fig. 7A) at the proximal growth plates of P28 humeri revealed a significant increase in the total number of YFP^+^ cells and in the number of YFP^+^ Osx^+^ cells by ∼70% each compared with the controls (Fig. 7C). At the growth plates undergoing active remodeling in humeri and femora, TRAP^+^ osteoclasts were detected but, compared with the control, the number of osteoclasts lining the cartilage erosion zone was decreased by ∼35% (Fig. 7B,D). Thus, the combination of increased chondrocyte-derived osteoblast precursor formation and decreased osteoclastogenesis observed at the growth plate undergoing remodeling is probably responsible for the increased BV/TV in the P28 *Ctnnb1*^GOFHTC^ limbs (see Fig. 3D, Fig. S3D).

## DISCUSSION

During the late phase of fetal development, remarkable remodeling processes occur in long bones – resorption of hypertrophic calcified cartilage, bone marrow cavity and trabecular bone formation. Our data show that the severe reduction of trabecular bone in mutants lacking β-catenin activity in late HTCs is the result of a combinatorial mechanism: decreased chondrocyte-derived osteoblast differentiation and increased recruitment of osteoclasts to the chondro-osseous front. The latter is due to local dysregulation of the Rankl:Opg ratio, and probably further supported by Opn and Mmp13 upregulation, as molecules potentiating osteoclast function (Franzen et al., 2008; Pivetta et al., 2011; Ross et al., 1993). Our results concerning *Rankl* regulation are consistent with previous findings (Golovchenko et al., 2013; Wang et al., 2014) and resemble the situation in osteoblasts (Holmen et al., 2005; Kramer et al., 2010; Sato et al., 2009; Spencer et al., 2006). Yet, the molecular mechanisms may differ between the cell types, being direct in osteoblasts (Spencer et al., 2006) and potentially indirect in chondrocytes via interference with a glucocorticoid receptor (GR)-dependent mechanism (Wang et al., 2014). However, the latter is questioned by the fact that the cartilage-specific *GR* (*Nr3c1*) knockout has no apparent growth plate or bone phenotype (Tu et al., 2014). Wang and colleagues used *Col2a1*-CreER in their study to inactivate β-catenin. This Cre line is also active in mesenchymal precursors of perichondrium-derived osteoblasts (Ono et al., 2014), which also require β-catenin. Hence, some of the phenotypic changes might be due to altered β-catenin activity in perichondrial-derived osteoblasts (Wang et al., 2014). In contrast to Wang and colleagues, we did not observe a decrease in height upon *Ctnnb1* loss or cellular disorganization upon β-catenin stabilization, suggesting that these changes correlate with β-catenin functions in *Col2a1*-expressing chondrocytes (Wang et al., 2014). Our hypothesis that increased *Rankl* expression in HTCs upon *Ctnnb1* deletion contributes substantially to the *Ctnnb1*^LOFHTC^ phenotype is supported by the reversal of the phenotype upon conditional removal of *Rankl* from HTCs in 50% of the specimens. The incomplete penetrance is likely to be explained by the fact that two *Rankl* alleles need to be recombined and Cre recombination efficiency at the cellular level varies between specimens (Nagy, 2000). Even in the four ‘complete’ *Ctnnb1*^GOFHTC^;*Rankl*^ΔHTC^ specimens the average BV/TV was at the lower end of the control range. This indicates that Rankl dysregulation is not the sole cause of the phenotype.

The 17-fold decrease in the number of osteoblasts derived by chondrocyte transdifferentiation together with the 18% decrease in perichondrial-derived Osx^+^ YFP^−^ precursor number in *Ctnnb1*^LOFHTC^ mutants are likely to contribute to the slightly lower BV/TV ratio of the ‘complete’ *Ctnnb1*^GOFHTC^;*Rankl*^ΔHTC^ specimens. Perichondrial-derived osteoblast precursors migrate along blood vessels into the bone marrow cavity (Maes et al., 2010). In the *Ctnnb1*^LOFHTC^ mutants blood vessels reach the chondro-osseous border and there is even hypervascularity, which is noticeable primarily in the diaphysis. The latter might be due to the transient increase in *Vegf* observed at E16.5, or the liberation of matrix-bound VEGF due to increased osteoclastic activity at the chondro-osseous front (Bergers et al., 2000; Huang et al., 2002). Thus, perichondrial-derived osteoblast precursors should be able to migrate normally, but due either to the lack of mineralized matrix, the remnants of late HTCs, or to other environmental changes, not all of them may find the right environment to survive or to differentiate along the osteoblastic lineage. While both osteoblastic populations decreased, the number of non-osteoblastic, chondrocyte-derived bone marrow cells increased by ∼18% in the *Ctnnb1*^LOFHTC^ mutants. The exact cellular nature of the YFP^+^ Osx^−^ population observed here and in previous lineage-tracing transdifferentiation studies has not yet been clarified (Park et al., 2015; Yang et al., 2014a,b; Zhou et al., 2014). Some of the YFP^+^ Osx^−^ cells in the *Ctnnb1*^LOFHTC^ mutants apparently remained in a more undifferentiated state, being positive for Runx2. Given that Wnt/β-catenin signaling plays an important role in the osteoblastogenesis versus adipogenesis lineage decision process of bone marrow cells (Bennett et al., 2005; Gambardella et al., 2011; Krishnan et al., 2006; Song et al., 2012), that some YFP^+^ Osx^−^ cells differentiated to adipocytes presented an intriguing possibility. However, the observed increase in adipocyte and adipocyte progenitor numbers in *Ctnnb1*^LOFHTC^ mutants was not due to a lineage switch of chondrocyte-derived osteoprogenitors. Instead, our data suggest that the FABP4^+^ progenitors originate from perichondrium-derived osteoprogenitors, explaining in part their reduction. Osteoblast precursors in the perichondrium that lack β-catenin activity differentiate into chondrocytes and express *Sox9* (Hill et al., 2005; Rodda and McMahon, 2006). Yet, the mechanism that blocks *Ctnnb1*-deficient cells from undergoing transdifferentiation appears to be distinct, as no alteration in Sox9 was observed. Neither altered apoptosis nor proliferation appears to cause the decrease in chondrocyte-derived osteoblastogenesis. Thus, the mechanism by which β-catenin controls this process is currently unclear.

In osteoblasts, *Opg* has been reported to be a direct, positively regulated β-catenin/Tcf target (Glass et al., 2005; Sato et al., 2009). However, this seems not to be the case in HTCs. In *Ctnnb1*^GOFHTC^ specimens, *Opg* transcript levels were not increased and only the most centrally located cells, which also expressed osteoblast markers, stained positively for Opg. Hence, β-catenin might only be able to regulate *Opg* in osteoblastic cells. Nevertheless, genetic loss of *Opg* reverted the *Ctnnb1*^GOFHTC^ phenotype to a certain extent, but only very late in embryonic development. Increased systemic Rankl levels in *Opg* mutants (Bennett et al., 2006) are likely to be responsible for the increase in osteoclast numbers. Yet, trabecular bone formation was abnormal and the mineralized HTC domains were still enlarged. The reason for the latter is probably multifactorial: despite *Opg* loss, *Rankl* expression by HTCs is probably still decreased and hence local osteoclastogenesis may not occur at the same rate as in wild type. This might contribute to the persistence of the enlarged mineralized hypertrophic zone in the *Ctnnb1*^LOFHTC^;*Opg^−^*^/−^ animals, as the hypertrophic zone is also increased in mutants with compromised osteoclastogenesis. In addition, *Opn* and *Mmp13* expression in late HTCs was still altered. As *Mmp13* mutants also display a widening of the HTC zone, the lack of *Mmp13* expression in late HTCs possibly contributes to the shortcomings of the phenotypic reversal (Inada et al., 2004).

Chondrocyte remodeling was absent in *Ctnnb1*^GOFHTC^ mice and late hypertrophic markers were not expressed, whereas the domains of immature markers such as *Ihh* and Sox9 were expanded. These data indicate that stabilization of β-catenin in HTCs, among others, delays terminal HTC differentiation*.* However, a dramatic expansion of mineralized HTCs was observed. Here, the centralmost chondrocytes started to express osteoblastic markers such as *Col1a1* and *Ppr* and produced Opg. We interpret this as an attempt by the cells to undergo osteoblastic transdifferentiation. Yet, these cells did not express osteocalcin, a marker of mature osteoblasts. Similar to the situation in periosteal osteoblast precursors, their attempt to mature might be stalled at an intermediate stage due to the high levels of stabilized β-catenin (Rodda and McMahon, 2006).

Although no true bone marrow cavity was formed in *Ctnnb1*^GOFHTC^ skeletal elements up to age P3, partial bone marrow cavity formation had occurred in 4-week-old mice. Interestingly, its occurrence was always associated with the more active growth plate (Farnum, 1994; Pritchett, 1991, 1992). In all postnatal growth plates, the number of new chondrocytes produced was equal to the HTCs lost, but in the more active growth plates these numbers were 2- to 3-fold higher (Wilsman et al., 1996). The factors responsible for this phenomenon are not yet known. Nevertheless, given that more chondrocytes are produced and turned over in the same amount of time in active growth plates, this means that chondrocytes spend less time in the hypertrophic zone (Wilsman et al., 1996). Consequently, *Col10a1*-Cre efficacy may decrease as the activity window becomes narrower and, as such, fewer cells recombine the *Ctnnb1* exon 3 floxed allele. As a result, *Rankl* would not be repressed in all HTCs and could support local osteoclastogenesis. Hence, bone marrow formation can reoccur and, due to increased chondrocyte transdifferentiation, trabecular bone formation is enhanced at these growth plates.

**Fig. 8.**
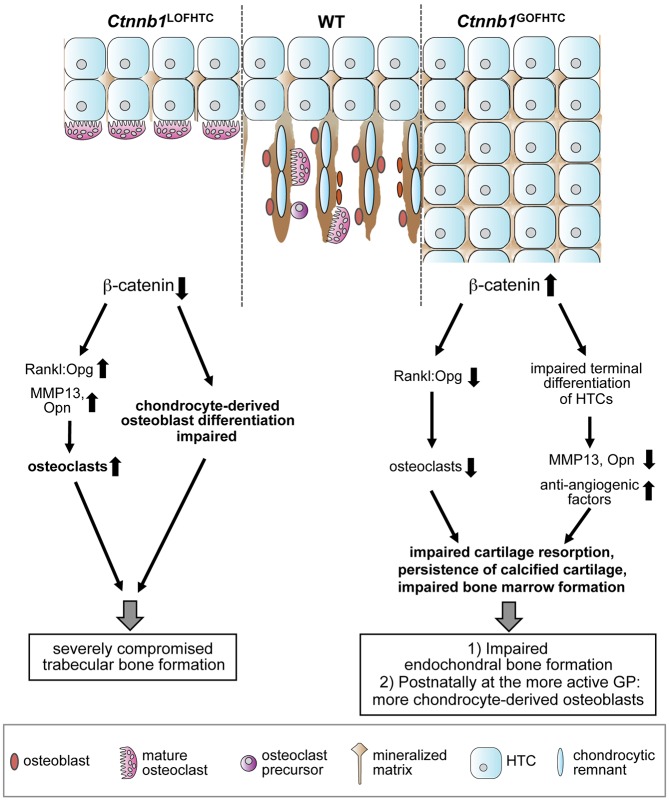
**Model summarizing the roles of** **β-catenin in late HTCs.** β-catenin locally regulates the Rankl:Opg ratio controlling osteoclastogenesis and negatively influences the expression of *Mmp13* and *Opn*. Inactivation of *Ctnnb1* from late HTCs leads to an increased Rankl:Opg ratio, increased expression of *Mmp13* and *Opn* and increased osteoclast numbers, but simultaneously affects the differentiation of osteoblasts derived from HTCs. Thus, this leads to a severe defect in trabecular bone formation. Stabilization of β-catenin results on the one hand in a decrease in the Rankl:Opg ratio and a subsequent reduction in osteoclast number and, on the other hand, affects the terminal differentiation of HTCs as reflected in the absence of *Mmp13* and *Opn* and their overall reduced expression, whereas anti-angiogenic factors are increased in expression. Thus, endochondral bone formation is severely impaired upon stabilization of β-catenin in HTCs owing to impaired cartilage resorption and bone marrow formation. GP, growth plate; WT, wild type.

In conclusion, we have shown that in late HTCs β-catenin has dual functions in trabecular bone formation: first, it locally regulates osteoclastogenesis by repressing the expression of the pro-osteoclastic factor Rankl, and second it is involved in the newly discovered transdifferentiation process by which HTCs give rise to osteoblast precursors (Fig. 8). As such, β-catenin is a key player in early trabecular bone formation during embryonic skeletal development.

## MATERIALS AND METHODS

### Mouse husbandry

The following strains were used: *Ctnnb1* floxed (Huelsken et al., 2001), *Ctnnb1* *lacZ* knock-in null (Huelsken et al., 2000), *Ctnnb1* exon 3 floxed (Harada et al., 1999), BAC-*Col10a1*-Cre (*Col10a1*-Cre, #1465 and #1421) (Gebhard et al., 2008), *Opg* (*Tnfrsf11b*) knockout (Mizuno et al., 1998), *Rankl* floxed (Xiong et al., 2011), *Rank* (*Tnfrsf11a*)^Δ*/*Δ^ (Hanada et al., 2009), and *Rosa26*-flox-Stop-flox-EYFP (*Rosa*^YFP^) (Srinivas et al., 2001). For breeding details see the supplementary Materials and Methods. For timed pregnancies, the plug date was considered to be E0.5. Genotyping was performed using previously published PCR protocols. Animals were sacrificed by cervical dislocation, embryos and newborns by decapitation. All mouse experiments were performed in accordance with local, institutional and national regulations and licenses (84-02.05.20.12.261).

### Microcomputed tomography (microCT) and histomorphometric analysis

Long bones were fixed for 24 h in 4% paraformaldehyde followed by two PBS washes and one wash in 70% ethanol for 24 h each. Prior to the scan, tissue was stored in 70% ethanol at 4°C and wrapped in Parafilm for the scan. Scans were performed using a SkyScan 1176 scanner (SkyScan, Kontich, Belgium). For scanning details see the supplementary Materials and Methods.

### Processing of specimens

For a detailed description of specimen processing for histology, immunohistochemistry and immunofluorescence see the supplementary Materials and Methods.

### Histology

Histological stainings, including Alcian Blue, Alcian Blue with von Kossa, Hematoxylin and Eosin (H&E) and TRAP were performed on 5 µm paraffin sections as described in the supplementary Materials and Methods.

### Immunohistochemistry and immunofluorescence

Staining for CD31, Opg, Osx, Sox9, β-catenin, collagen type II, DIPEN, Runx2, YFP, Ki67, FABP4, endomucin, cleaved caspase 3 and TUNEL was performed on deparaffinized and rehydrated 5 µm sections as described in the supplementary Materials and Methods.

### Alkaline phosphatase-based and double fluorescent *in situ* hybridization

*In situ* hybridization using digoxigenin (DIG)-labeled RNA probes was performed according to Murtaugh et al. (1999). Double-fluorescent *in situ* hybridization on paraffin sections was performed using antisense biotin-labeled *Col1a1* probe and DIG-labeled *Col10a1* or *Ppr* probes according to Taschner et al. (2008). Probes for *Col10a1*, *Col1a1*, *Ihh*, *Osc* (Hill et al., 2005), *Mmp13* (Yamagiwa et al., 1999), *Ppr* (Spater et al., 2006b), *Opn*, *Mmp9*, *Vegfa* (Golovchenko et al., 2013), *Catk* (Bozec et al., 2008) and *Trap* (Lubberts et al., 2002) have been published previously and are available upon request.

### RNA isolation from FACS-sorted primary HTCs and total skeletal elements

RNA was isolated from dissociated whole P0 skeletal elements or from YFP-positive cells isolated from E16.5 skeletal elements using the RNeasy Kit (Qiagen) according to the manufacturer's instructions, including a DNase I treatment. RNA concentration was determined by OD. For details, see the supplementary Materials and Methods.

### cDNA synthesis

For first-strand cDNA synthesis, 0.5-1 µg total RNA was reverse transcribed using oligo(dT) primers. For qPCR analysis, cDNA was diluted 1:10. For details see the supplementary Materials and Methods.

### Real-time PCR analysis

First-strand cDNA (2-3 µl of 1:10 diluted) was mixed with either Fast Start SBG Master Mix (Roche, #04673484001) or SYBR Premix Ex Taq II (TaKaRa, #RR820Q). Gene expression was monitored using a Bio-Rad CFX96 cycler. For details see the supplementary Materials and Methods and Table S1 for primer sequences.

### Image acquisition

Histological images were acquired using either a Zeiss AxioPlan2 equipped with a Leica DFC320 3.45 µm pixel color camera or a Zeiss AxioImager.M2 equipped with a Zeiss AxioCam MRc 6.45 µm color camera. Immunofluorescent images were acquired using an AxioImager.M2 equipped with an ApoTome.2 and an AxioCam MRm 6.45 µm monochromatic camera using Zen software (all Zeiss).

### Statistical analysis

Statistical analysis was performed by two-tailed, unpaired Student's *t*-test using GraphPad Prism 6.0. Data are displayed as mean ±s.d.
